# Strong inflammatory responses and apoptosis in the oviducts of egg-laying hens caused by genotype VIId Newcastle disease virus

**DOI:** 10.1186/s12917-016-0886-2

**Published:** 2016-11-15

**Authors:** Ruiqiao Li, Kangkang Guo, Caihong Liu, Jing Wang, Dan Tan, Xueying Han, Chao Tang, Yanming Zhang, Jingyu Wang

**Affiliations:** College of Veterinary Medicine, Northwest A&F University, Yangling, Shannxi 712100 China

**Keywords:** Genotype VIId NDV, Oviduct, Apoptosis, Inflammatory responses, Lymphocyte infiltration, Egg-laying hens

## Abstract

**Background:**

Newcastle disease virus (NDV) can cause serious damage to the reproductive tracts of egg-laying hens and leads to egg production and quality reduction. However, the mechanism of severe pathological damage in the oviducts of egg-laying hens after NDV infection has not been fully elucidated. In this study, the correlation between the primary pathological lesions and viral load in the oviducts of egg-laying hens infected with the velogenic genotype VIId NDV strain was evaluated by pathological observation and virus detection. Subsequently, apoptosis, the expression of immune-related genes and lymphocyte infiltration into the infected oviducts were determined to explore the potential causes of the pathological changes.

**Results:**

A higher viral load and severe tissue lesions and apoptotic bodies were observed in the oviduct of NDV-infected hens compared with the control. Immune-related genes, including TLR3/7/21, MDA5, IL-2/6/1β, IFN-β, CXCLi1/2, and CCR5, were significantly upregulated in the magnum and uterus. IL-2 presented the highest mRNA level change (137-fold) at 5 days post infection (dpi) in the magnum. Infection led to CD3^+^CD4^+^ and CD3^+^CD8α^+^ lymphocyte infiltration into the magnum of the oviduct. A higher viral load was found to be associated with pathological changes and the elevated expression of proinflammatory cytokines in the NDV-infected hens.

**Conclusions:**

Our results indicate that the severe lesions and apoptosis in the oviducts of egg-laying hens caused by genotype VIId NDV strains are associated with the excessive release of inflammatory cytokines, chemokines and lymphocyte infiltration, which contribute to the dysfunction of the oviducts and the decrease of egg production in hens.

## Background

The Newcastle disease virus (NDV) is highly contagious and widespread among avian species and causes severe economic losses in domestic poultry, especially chickens [1]. It does not only cause the death of chickens but also causes serious damage to the reproductive tracts of egg-laying hens. This leads to an overall decrease in egg production and increases the percentage of deformed, sand-shelled, and soft-shelled eggs [2]. Since the 1950s, vaccination has been applied for the prevention and control of this disease in many countries, including China. However, it is still enzootic in some areas and is recognized as a major disease that affects poultry [3]. The genotype VII NDV is dominant in Asia and poses a serious threat to its poultry industry [4–6]. Previous studies have revealed that the significant oviduct inflammation and decline of egg production are caused by the variance [2, 7]. However, the inflammatory effects and apoptosis of local oviduct of the virulent NDV genotype VIId strains were still unclear, which may lead to oviduct dysfunction and drop in egg production.

Generally, host-virus interactions caused by NDV include a complex interplay of molecular pathways directed by the host to prevent viral replication [8, 9]. These involve intense inflammatory responses in the form of a cytokine storm that are initiated inside infected cells or tissues following viral replication and result in excessive cellular apoptosis and tissue damage. In vivo studies suggested that NDV initiated strong immune responses in the spleen, lymph nodes and peripheral blood of infected chickens, where the mRNA expression levels of proinflammatory and cytokines/chemokines were significantly upregulated [9–12]. Strong innate immune response and cell death were observed in chicken splenocytes infected with genotype VIId NDV [13]. The cytopathic effect (CPE) and apoptosis were observed in NDV infected cells and viral replication and caspase activation were detected [14–16]. Studies investigating the mechanism underlying severe pathological damages in the oviducts of egg-laying hens after NDV infection are limited. Furthermore, the reproductive tract is a target of both the vaccine strain and field isolates of NDV [7], so the innate immunity roles of oviduct played a critical role in viral pathogenesis, especially, the inflammatory effects and attribution of different immune cells and immune molecules.

In this study, a severe oviduct tissue damage of egg-laying hens were observed in the genotype VIId NDV strain infected hens. This damage was associated with high levels of virus replication, a strong innate immune response/inflammatory response and lymphocyte infiltration. These responses contributed to oviductal dysfunction and the decline of egg production, which played a critical role in viral pathogenesis.

## Results

### Clinical signs and histopathological changes

Obvious ND-related clinical signs, including ruffled feathers, depression, and drowsiness, were observed in NDV-infected birds 2 dpi. At 3 dpi, the infected hens showed typical clinical signs, including dyspnea, depression and greenish feces. The first bird died of NDV infection 3 dpi. At 4 dpi, Four infected hens died from viral challenge-associated causes. The most severe deaths occurred 5 dpi, when seven infected birds died. Furthermore, egg production of the NDV-infected hens started to plunge by approximately 40% on approximately 5 dpi, whereas the controls maintained approximately 90% egg production (Table 1). From 7 dpi, no hens died spontaneously or was moribund, and clinical signs and egg production of the remaining hens began to abate. Upon examination of the gross lesions, the experimental group hens showed proventricular hemorrhage, tracheorrhagia, brain edema and dissolved follicles, while the oviducts showed edema. Hens in the control group did not show any clinical signs or gross lesions throughout the procedure.

**Table 1:** Tissue processing and egg production changes in chickens infected with the velogenic genotype VIId NDV

Day	Control group			Experimental group				
	Total	Sample	Laying rate%	Total	Mortality	Autopsy	Sample	Laying rate %
0	45	0	91.1 (41/45)	55	0	0	0	90.9 (50/55)
1	45	5	87.5 (35/40)	55	0	5	5	88.0 (44/50)
3	40	5	88.6 (31/35)	50	1	5	5	66.7 (30/45)
4	35	0	91.4 (32/35)	45	4	4	0	48.8 (20/41)
5	35	5	90.0 (27/30)	41	7	7	5	38.2 (13/34)
6	30	0	90.0 (27/30)	34	3	3	0	41.9 (13/31)
7	30	5	88.0 (22/25)	31	1	5	5	46.2 (12/26)
9	25	5	90.0 (18/20)	26	0	5	5	52.4 (11/21)
11	20	5	86.7 (13/15)	21	0	5	5	56.3 (9/16)
15	15	5	90.0 (9/10)	16	0	5	5	72.7 (8/11)

**Fig. 1:**
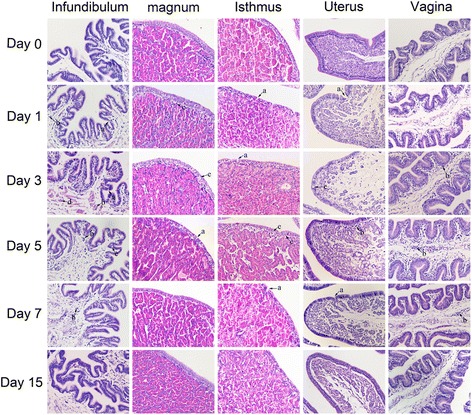
Histopathology of the oviducts stained with hematoxylin and eosin (400×). (A) Cilia loss. (B) Lymphocyte infiltration. (C) Heterophil infiltration. (D) Pink-stained material in the infundibulum. (E) Mucosal epithelial necrosis

Histopathological examinations of infundibulum, magnum, isthmus, uterus, and vagina are shown in Fig. 1. The tissues of the laying hens of the control group appeared normal. On 1 dpi, there were scattered necrotic areas in the mucosal epithelia and lymphocyte infiltration into the infundibulum, magnum, isthmus, and uterus. Occasionally, heterophils were found in the lamina propria of the magnum. On 3 dpi, the uterus presented marked edema. Severe mucosal epithelial necrosis and numerous lymphocytes were seen in the vagina. On 5 dpi, the degree of the lesions in the isthmus was elevated, while mucosal epithelial necrosis, heterophil infiltration, and edema were also observed. Moreover, a large number of lymphocytes were also observed in the vagina. On 7 dpi, the lesions in the infundibulum became less severe. However, heterophil infiltration and mucosal epithelial necrosis persisted. On 15 dpi, some lymphocytes were found in the infundibulum. No significant changes were observed in the control group.

### Viral loads in oviduct

**Fig. 2:**
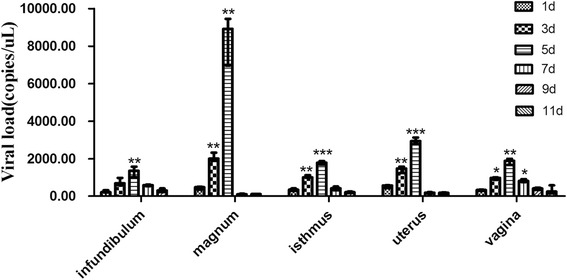
NDV viral load in oviduct. Primers targeting the M gene of NDV were used to detect virus replication in the oviducts of hens infected with the velogenic genotype VIId NDV strain by quantitative RT-PCR assay with three replications. According to our standard curve, the correlation coefficient (*R* ^2^) was 0.9979 with a slope value of -3.3749 (y = -3.3749 × + 37.202)

To evaluate differences in viral replication in the oviduct of egg-laying hens after NDV infection, we determined the transcriptional levels of the viral matrix (M) gene in the oviducts of hens infected with the velogenic genotype VIId NDV strain by quantitative RT-PCR assay. We detected the viral load from the infundibulum, magnum, isthmus, uterus, and vagina taken at 1, 3, 5, 7 and 9 dpi. The level of viral RNA is shown in Fig. 2. The viral load was significantly increased in the infundibulum, magnum, isthmus, uterus, vagina and reached the peak at 5 dpi, gradually decreased at 7, 9 and 11 dpi. The magnum presented the highest amount of virus load (8918.6 copies/ml), followed by the uterus (2934.6 copies/ml), vagina (1890 copies/ml), isthmus (1789 copies/ml), and infundibulum (1358 copies/ml) (Fig. 2). No virus was found in healthy oviducts.

### Virus distribution along the oviductal segments

**Fig. 3:**
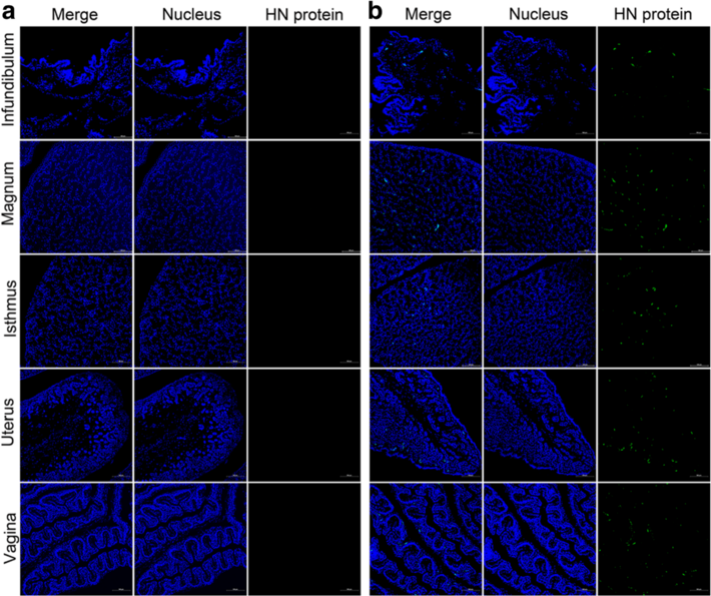
Detection of the NDV HN protein in the oviducts by IFA at 5 dpi. Distribution of the NDV HN protein was detected by staining with a mouse anti-HN monoclonal antibody and fluorescein isothiocyanate (FITC)-conjugated goat anti-mouse secondary antibody (green fluorescence); the nuclei were stained with Hoechst 33342 (blue fluorescence) (400×). **a** Control hens. **b** Infected hens

Indirect immunofluorescence assays were conducted to confirm the distribution of the NDV HN protein in the oviducts at 5 dpi. Positive staining was not observed in any part of the oviducts of the control hens. However, HN proteins were observed in the glandular epithelial cells of the lamina propria and found scattered in the submucosa of the infundibulum, magnum, and isthmus. Conversely, the HN proteins were present in the submucosa and scattered in the lamina propria in the uterus. In the vagina, they were located in the mucosal epithelium (Fig. 3).

### Apoptosis in the oviducts of the infected hens

**Fig. 4:**
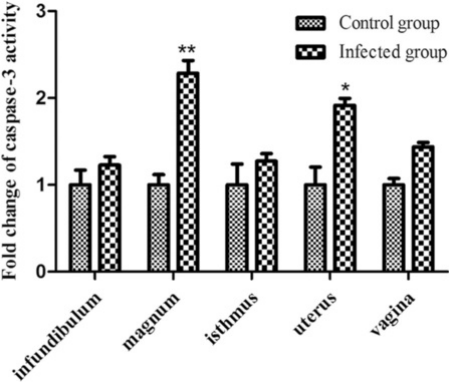
Caspase-3 activity in the oviducts following NDV infection. Total protein was extracted from infected and uninfected chickens 5 dpi (*n* = 3 per group), and caspase-3 activity was detected using a specific detection kit. The data represent the mean ± SEM of three chickens. Each column represents the fold change of caspase-3 activity obtained by comparison with the data from uninfected chickens. *P <0.05, **P <0.01, ***P <0.001

**Fig. 5:**
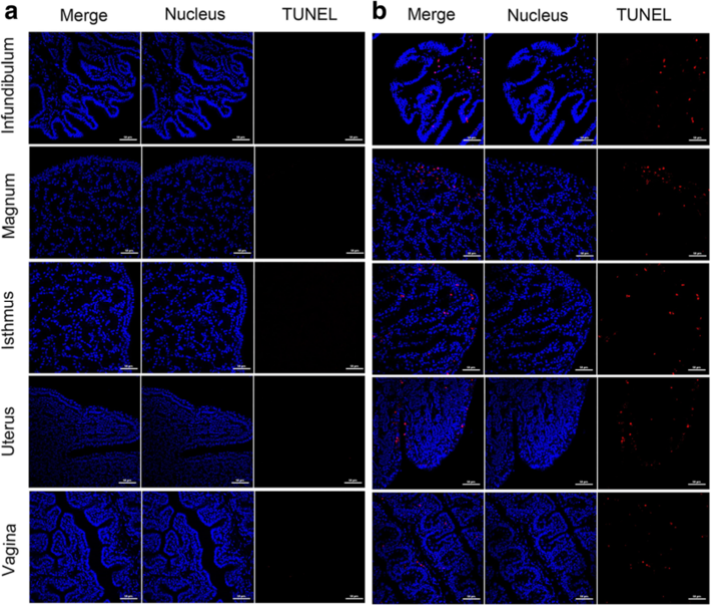
TUNEL staining of the oviduct in NDV-infected hens. Oviduct tissues were collected 5 dpi. The nuclei of oviduct sections from virus-free and NDV-infected hens were stained with Hoechst 33342; a red color indicates the positive staining of apoptotic cells (400×). Oviductal segments from virus-free hens served as the negative control. **a** Control hens. **b** Infected hens

In this study, caspase-3 activity and TUNEL assays were employed to detect apoptosis in the oviducts of the infected hens on 5 dpi. The caspase-3 activities in the magnum and uterus were found higher compared to the control group (2.41- and 1.98-fold increases, respectively) (Fig. 4). The results of the TUNEL assays revealed that NDV induced apoptosis in the lamia propria of the infundibulum and vagina (Fig. 5). In the magnum, isthmus, and uterus, apoptotic cells were observed in both the lamia propria and the mucosa.

### Expression of TLRs, MDA5, cytokines, and chemokines

**Fig. 6:**
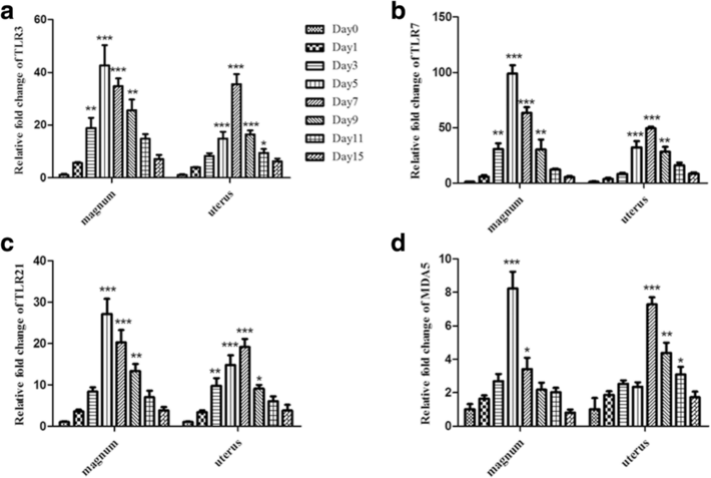
mRNA expression levels of the TLRs and MDA5 in the magnum and uterus of laying hens infected with NDV. Total RNA was extracted from the infected and control groups (*n* = 3 per group). The TLRs and MDA5 were quantified using appropriate primers specific to the chicken and SYBR Green-based real-time (RT) PCR. The data represent the mean ± standard error of the mean (SEM) for three chickens. Panels **a**, **b**, **c**, and **d** depict the results for TLR3, TLR7, TLR21, and MDA5, respectively, in the magnum and uterus. Statistical analysis was performed by comparison with the healthy chickens. *P <0.05, **P <0.01, ***P <0.001

To estimate the inflammatory response in the oviducts of the infected hens, a real-time quantitative PCR assay was used to quantify the expression profiles of immune-related genes in the magnum and uterus of the oviduct of SPF egg-laying hens infected with genotype VIId NDV on 1, 3, 5, 7, 9, 11 and 15 dpi. The mRNA levels of TLR3, 7, and 21 were altered in the oviducts of the infected laying hens (Fig. 6) and were upregulated in the magnum and uterus tissues. The mRNA expression levels in the magnum segments peaked at 5 dpi (42.6-, 98.9-, and 27.1-fold, respectively), while in the uterus they peaked on 7 dpi. Moreover, TLR expression levels in the magnum were significantly greater compared to the uterus. This result suggests that the magnum segments were most sensitive to NDV. Another intracellular pattern recognition receptor (PRR), MDA5, was also upregulated, with its highest fold changes of 8.23 and 7.29 detected in the magnum and uterus, respectively (Fig. 6d).

**Fig. 7:**
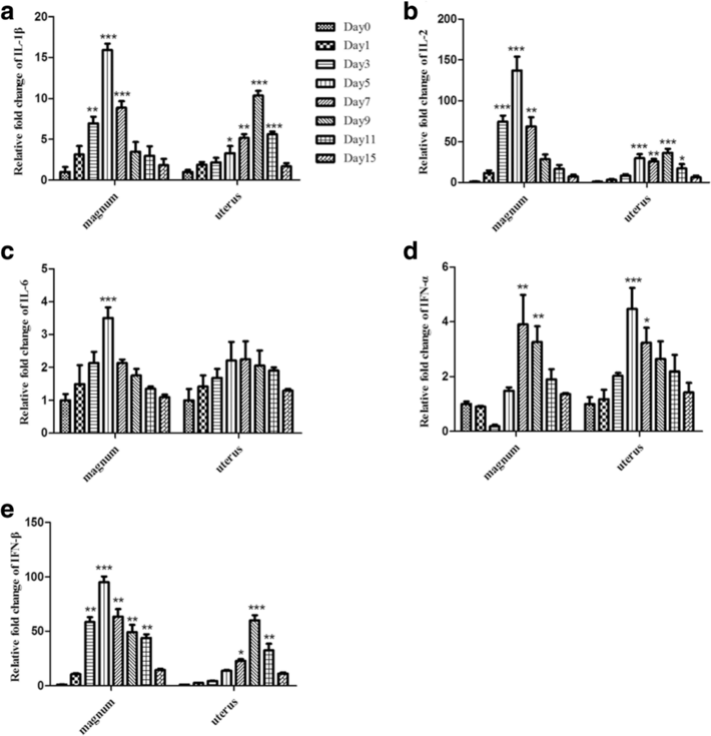
Expression levels of inflammatory cytokines in the magnum and uterus of laying hens infected with NDV. IL-1β/2/6 and IFN-α/β were quantified using SYBR Green-based RT-PCR. Panels **a**, **b**, **c**, **d** and **e** depict results for IL-1β, IL-2, IL-6, IFN-α, and IFN-β, respectively, in the magnum and uterus. Statistical analysis was performed by comparison with uninfected chickens. *P <0.05, **P <0.01, ***P <0.001

The expression levels of IL-2/6/1β and IFN-α/β were also examined at the transcript level (Fig. 7). IL-2/6/β and IFN-β were significantly upregulated at two instances during the infection process, but the ranges of upregulation were different. IL-2 presented the highest mRNA level change of 137-fold on 5 dpi (Fig. 7b), followed by IFN-β (95.1-fold) and IL-1β (15.9-fold). In contrast, IFN-α was downregulated by 0.19-fold 3 dpi and subsequently upregulated by 1.78-fold 5 dpi in the magnum, but not in the uterus (Fig. 7d). Furthermore, IFN-α expression was significantly upregulated and peaked 5 dpi with a 4.56-fold increase in the uterus (Fig. 7d).

**Fig. 8:**
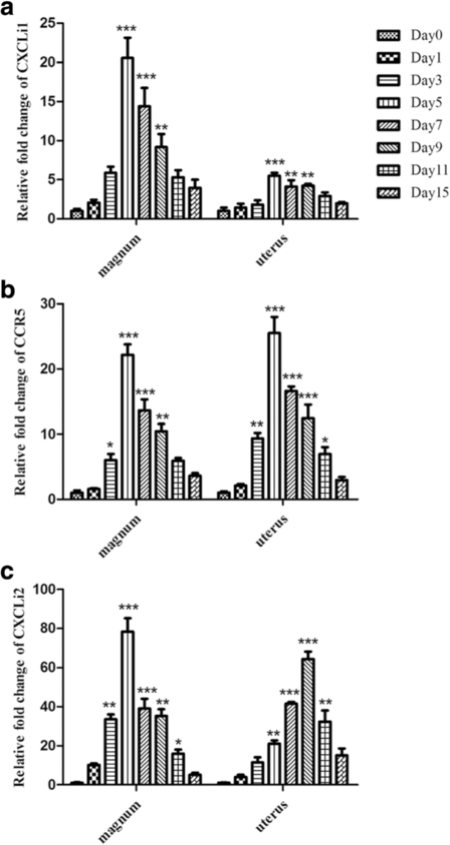
mRNA expression levels of CXCLi1, CCR5, and CXCLi2 in the magnum and uterus of laying hens infected with NDV. The data represent the mean ± SEM of three chickens. Panels **a**, **b**, and **c** depict the results for CXCLi1, CCR5, and CXCLi2, respectively, in the magnum and uterus. Statistical analysis was performed by comparison with the uninfected chickens. *P <0.05, **P <0.01, ***P <0.001

We examined three chemokines (CXCLi1/2 and CCR5) (Fig. 8). The mRNA expression level of CXCLi1 was increased in the magnum throughout the infection process, reaching its peak of 20.5-fold on 5 dpi. It was also significantly upregulated (5.48-fold) in the uterus on 5 dpi (Fig. 8a). The chemokine receptor for CXCLi1 (CCR5) was also upregulated during the infection process. This upregulation was higher than CXCLi1 in the magnum (22.1-fold) and uterus (25.5-fold) (Fig. 8b). CXCLi2 was also significantly upregulated and peaked at 5 dpi with a 78.3-fold increase in the magnum (Fig. 8c).

### 8Dynamic changes in CD3^+^CD4^+^ and CD3^+^CD8α^+^ lymphocytes in the oviducts

**Fig. 9:**
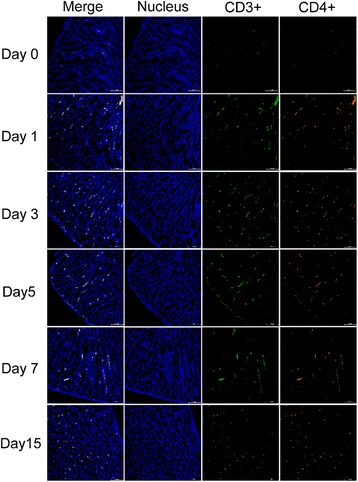
Infiltration of CD3^+^CD4^+^ cells in the magnum of NDV-infected laying hens. Egg-laying hens were inoculated with the NDV at a dose of 10^6^ EID_50_, and the magnums of the oviducts were collected 0, 1, 3, 5, 7, 9, 11, and 15 dpi. The nuclei were stained with Hoechst 33342 (400×). The CD3^+^ lymphocytes were detected by a FITC-conjugated mouse anti-chicken monoclonal antibody. A green color indicates positive staining. The CD4^+^ lymphocytes were detected by a CY5-conjugated mouse anti-chicken monoclonal antibody. A red color indicates positive staining

**Fig. 10:**
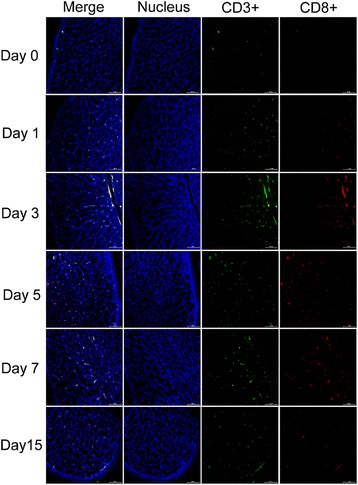
Infiltration of CD3^+^CD8α^+^ lymphocytes in the magnum of NDV-infected laying hens. The nuclei were stained with Hoechst 33342 (400×). The CD3^+^ lymphocytes were detected by a FITC-conjugated mouse anti-chicken monoclonal antibody. A *green* color indicates positive staining. The CD8^+^ lymphocytes were detected by a RPE-conjugated mouse anti-chicken monoclonal antibody. A salmon color indicates positive staining

To study the dynamic changes in the CD3^+^CD4^+^ and CD3^+^CD8α^+^ lymphocytes in the oviducts of hens after NDV infection, we selected the magnum, which was the segment with the highest level of viral replication and mRNA expression of immune-related factors, for further study. The accumulation of CD3^+^CD4^+^and CD3^+^CD8α^+^ lymphocytes in the magnum was greater in the experimental than in the control group throughout the course of infection (Figs. 9 and 10). The CD3^+^CD4^+^ and CD3^+^CD8α^+^ lymphocytes began to accumulate 1 dpi and peaked 5 and 7 dpi, respectively (Figs. 9 and 10).CD3^+^CD4^+^ and CD3^+^CD8α^+^ lymphocytes were distributed in the lamina propria of the magnum. Moreover, CD8α^+^ cells were recruited into the magnum more deeply than the CD4^+^ cells. The frequency of CD4^+^ cells in the lamina propria was significantly higher compared to the bottom of the mucosal epithelium. In contrast, the CD8α^+^ cells localized predominantly at the bottom of the mucosal epithelium. Sporadic distribution of these lymphocytes was detected in the oviducts in the control birds.

## Discussion

Newcastle disease is a highly contagious viral disease that causes serious economic damage to the poultry industry [4]. The velogenic genotype VII NDV has been documented as the predominant epidemic genotype and has been responsible for frequent outbreaks on vaccinated farms in China and other Asian countries since 1990 [17, 18]. The strain used in this study was isolated from a vaccinated farm. We observed that the significant clinical signs, pathological changes, and apoptosis induced by velogenic genotype VIId NDV infection contributed to oviduct dysfunction and the decline of egg production in the hens. Our findings also indicated that the severe tissue damage in the reproductive tract was associated with high viral replication, apoptosis, an intense inflammatory response, and lymphocyte infiltration.

In this study, examination of the viral load confirmed that NDV replication occurred in the oviducts of egg-laying hens. At 5 dpi, when the viral load was higher than 1, 3, 7,11 and 15 dpi, the histopathology were more severe, which may be due to the higher viral load in oviduct at these days. Therefore, the efficiency of viral replication in oviduct of hens appears to be associated with the elevated expression of proinflammatory cytokines in the NDV-infected hens. Furthermore, positive staining for the NDV HN protein in the oviducts revealed that the infected cells were glandular epithelial and mucosa cells. This finding was consistent with the presence of apoptotic cells in the five oviductal segments observed in the current study. We also observed the invasion of inflammatory cells and necrocytosis in the mucosal epithelia in five segments (Fig. 1). We hypothesized that the lesions aroused by NDV infection in the five segments might lead to the poor performance in egg-laying hens, thereby resulting in decreased egg production.

Apoptosis is a critical mechanism of NDV-induced tissue lesions, and caspase-3 plays a critical role in the execution of the apoptotic process [19, 20]. In our study, caspase-3 activity was significantly high in the NDV-infected layers, which was consistent with the results of a previous study [16]. Furthermore, our TUNEL assays also revealed apoptosis in the five oviduct segments upon NDV infection. Collectively, these results indicate that the oviductal tissues undergo cellular apoptosis both in the early and late stages of NDV infection.

We examined whether pattern recognition receptors (PRRs), inflammatory cytokines and chemokines in the oviduct exhibited altered expression in response to NDV infection. TLRs and MDA5 belong to different PRRs that recognize viral nucleic acids and are central to host antiviral defenses. TLRs are the most important family of PRRs and activate the MyD88-dependent pathway to produce cytokines, MHC molecules, and chemokines. In turn, these molecules trigger an appropriate immune response to eliminate the invading pathogens [21, 22]. In birds, the TLRs 3, 7, and 21 play major roles in the induction of antiviral IFN-β during such infections [23, 24]. In our study, the transcriptional levels of TLR3/7 were significantly increased post-NDV infection, suggesting the anti-NDV roles of these molecules.

Cytokines and chemokines are major mediators of the host immune response during viral infection [25]. NDV infection induced strong pro-inflammatory responses that played an important role in viral pathogenesis. The IL-2/6/1β and IFN-β mRNA expression levels were increased in the oviductal tissues of NDV-infected hens; our data are consistent with previous findings [9–12]. The expression of the IL-2 gene in the peripheral blood of F48E9 NDV-infected chickens in a previous study fluctuated and did not exhibit a significant difference compared with the control group during the experimental period [26]. In contrast, the expression of IL-2 in our study increased rapidly and presented the highest mRNA level change (137-fold) at 5 dpi. IFN-α belongs to the type I interferon family and can be produced in most cells after virus infection, whereupon it localizes to the site of viral infection and inhibits viral replication. In this study, IFN-α experienced a decline in the magnum of hen oviducts 3 dpi; the same phenomenon was reported in another study that demonstrated that the expression of IFN-α experienced a sharp decline in the peripheral blood of F48E9 NDV-infected chicken 7 dpi [12], which might contribute to rapid viral replication. The highest levels of expression of the examined genes corresponded with the significant clinical signs and the inflammatory responses in the oviductal tissues. In the chicken, there are two syntenic genes (CXCLi1 and CXCLi2) that mainly chemoattract heterophils and monocytes, respectively [27]. Interestingly, although both the CXCL chemokines were upregulated following NDV infection in this study, the CXCLi2 mRNA expression levels were higher compared to CXCLi1. The upregulation of CXCLi1, CXCLi2, and CCR5 might potentially attract immune cells, thereby modulating cellular immunity.

Cell-mediated immunity is a specific adaptive immune response mediated by T lymphocytes. It is suggested to be an important factor in the development of protection in chickens vaccinated against NDV and viral clearance [28–30]. T lymphocytes are classified according to their expression of cell surface proteins. The CD3 molecule is the surface marker for mature lymphocytes; most CD4^+^ lymphocytes are helper cells, while CD8^+^ lymphocytes are cytotoxic cells [31]. In this study, immunofluorescence staining in the magnum demonstrated lymphocyte infiltration. CD3^+^CD4^+^ and CD3^+^CD8α^+^ lymphocytes were distributed in the lamina propria and mucosal epithelium of the magnum. Furthermore, the CD3^+^CD8α^+^cells were located and infiltrated deeper into the bottom of the mucosal epithelium compared with the CD3^+^CD4^+^ cells. Thus, infiltration of CD3^+^CD8α^+^ lymphocytes may be responsible for the aggravated tissue lesions and mediate viral clearance. A previous study suggested that T cell recruitment in association with the expression of proinflammatory cytokines and the CXCLi2 chemokine were upregulated in response to LPS in the lower part of the oviduct [32], our data are consistent with their findings. The high degree of lymphocyte infiltration in the NDV-infected chickens might contribute to the elevated oviductal damage observed during the early stage of infection.

Overall, NDV is a continuing threat to the poultry industry. Our findings revealed that the oviducts of laying hens were potential target tissues for NDV infection. Our results suggest that the strong inflammatory responses and apoptosis induced by NDV infection lead to severe local damage in the oviducts of egg-laying hens. We also measured the eggshell and albumin quality in another study, and our results suggested that NDV caused eggshell thinning and decreases in the expression of ovalbumin (OVA) and calcium-binding protein D28K (CaBP-D28k) (data not shown), all of which may contribute to the reduced egg production. Given the findings that the magnum is the most sensitive segment to NDV, this sensitivity may contribute to the poor albumen quality of eggs laid by NDV-infected birds. Further studies are needed to determine the mechanisms underlying the apoptosis and immune responses in infected hen oviductal cell lines in vitro.

## Conclusions

Infection with the velogenic genotype VIId NDV triggered apoptosis and production of cytokines and chemokines in oviduct, accompanied by inflammatory responses. It further led to infiltration of CD3 + CD4+ and CD3 + CD8α + lymphocytes in the segments of the oviduct. Our results indicate that the severe lesions in the oviducts of egg-laying hens caused by genotype VIId NDV strains is associated with excessive release of inflammatory cytokines and chemokines and lymphocytes infiltration, which contribute to the dysfunction of oviducts and the decline of egg production in hens.

## Methods

### Virus

The velogenic genotype VIId NDV strain (NDV/Chicken/TC/9/2011) was isolated from Shanxi province, China, in 2011 [33]. The mean death time (MDT) and intracerebral pathogenicity index (ICPI) were previously determined to be 48 h and 1.69, respectively . The virus was cultured in 10-day-old specific-pathogen-free (SPF) embryonated chicken eggs (Green Biological Engineering Co., Yangling, China).

### Animals and experimental design

One hundred forty-week-old specific-pathogen-free (SPF) White Leghorn egg-laying hens (Green Biological Engineering Co., Yangling, China) were housed in isolators under negative pressure with food and water provided ad libitum. They were randomly divided into the control group and experimental group. The forty-five hens of control group were used as the NDV-free controls. The fifty-five hens in the experimental group were infected with a total of 0.5 mL (10^6^ median embryo infective doses) of the velogenic genotype VIId NDV strain through combined intraocular and intranasal routes. The infected hens were monitored for clinical signs of disease and egg production. All animals were checked at least twice daily to ensure their health and welfare.

Animals displaying severe clinical distress were euthanized with intravenous sodium pentobarbital at a dose of 100 mg/kg. Five birds from each group was humanely euthanized on days 1, 3, 5, 7, 9, 11 and 15 post-infection (dpi), and various parts of the oviduct were sectioned. Birds that died as a result of infection were included with the sampled birds on the indicated days. Birds that died on other days were observed by autopsy only. The criteria for euthanasia were somnolence, akinesia and dyspnea. Tissue processing and egg production changes were shown in Table 1. All experiments were performed in laboratories with biosafety level 2 facilities.

Tissue samples of the infundibulum, magnum, isthmus, uterus, and vagina from the control and experimental groups were collected immediately and placed on ice. Some parts of the tissues were preserved in liquid nitrogen for SYBR Green-based real-time (RT)-PCR, and others were fixed in 10% phosphate-buffered formalin for more than 24 hs [34]. The blocks were sectioned at 5-μm thicknesses for histopathological lesion observation, terminal deoxnuceotidyl transferase-mediated d-UTP biotin nick end labeling (TUNEL) staining, hemagglutinin neuraminidase (HN) protein detection, and CD3^+^CD4^+^ and CD3^+^CD8α^+^ lymphocyte detection by immunofluorescence.

### Histopathological examination

The infundibulum, magnum, isthmus, uterus, and vagina were collected and fixed in 10% phosphate-buffered formalin, embedded in paraffin wax and cut into sections using routine protocols. Next, the paraffin sections were stained with hematoxylin and eosin, and the tissue changes were observed under a light microscope.

### Detection of viral loads in the oviducts

The SYBR Green I (TransGen biotech, China) RT-PCR assay was used to detect the viral loads in the infundibulum, magnum, isthmus, uterus, and vagina. The M gene of NDV was used as the target gene and as a reference for the expression analyses. The primers used are as follows: Forward: 5′-ATCCGAAGAGCCCGTTAG-3′ (3939-3956) and Reverse: 5′-ACATCACTGAGCCCGACA-3′ (4113-4096). Total RNA was extracted from the reproductive tract tissues using the TRIzol reagent (Takara Biotechnology, Japan). The concentration of RNA in each sample was measured spectrophotometrically at 260 nm versus 280 nm. Next, the RNA was reverse-transcribed using the EasyScript First-Strand cDNA Synthesis SuperMix (TransGen Biotech) according to the manufacturer’s instructions. Construction of standard curve was done according to the procedure of [34].

### Detection of the viral hemagglutinin-neuraminidase protein in the oviduct by indirect immunofluorescence

The paraffin sections of the oviducts were examined by IFA to detect the viral HN protein. Briefly, the tissue sections were deparaffinized with xylene for 10 min, followed by hydration through a graded series of alcohol to xylol (100%, 95%, 90%, 80%, and 70%) for 3 min at each step. Then, the sections were blocked with 5% BSA for 1 h at 37 °C. Next, the sections were incubated with1 mg/ml mouse anti avian monoclonal antibody specific for HN of NDV (Santa Cruz Biotechnology) in PBS overnight at 4 °C. After washing thrice with PBS, the sections were incubated with FITC-conjugated goat anti-mouse IgG (Santa Cruz Biotechnology, 1:400) in PBS for 1 h at 37 °C, and then washed thrice with PBS once again and counter stained with Hoechst 33342 (Sigma, USA) for 5 min. Finally, the treated sections were observed under a confocal laser scanning microscope (Nikon, Japan).

### Determination of apoptosis in the oviduct

#### Detection of caspase-3 activity

A total of 0.1 g of oviduct tissue was cut into small pieces, added to 1 mL of radio immunoprecipitation assay lysis buffer, and homogenized in a glass pestle on ice. The tissue homogenates were transferred into 1.5 mL centrifugetubes and incubated on ice for 5 min. Then, they were centrifuged at 20,000 rpm for 10 min, and the supernatants were collected into pre-cooled centrifuge tubes. The caspase-3 enzyme activity was measured using the detection kit (KeyGen Biotech), and its concentration was determined by the Bradford Protein Assay Kit (Beyotime Institute of Biotechnology, Nanjing, China).

#### Quantification of apoptosis by the TUNEL assay

Apoptosis was examined using the one-step measurement of terminal deoxnuceotidyl transferase mediated d-UTP biotin nick end labeling (TUNEL) in the oviducts of the infected hens on 5 dpi. Tissue sections were deparaffinized and rehydrated through an alcohol gradient then rinsed in deionized water. The following steps corresponded to the standard operation staining procedures provided by manufacturer (Vazyme Biotech, Nanjing, China).

### Quantification of TLRs, MDA5, and proinflammatory cytokines by RT-PCR

Total RNA extraction was performed as described in the detection of viral loads in the oviducts section. RT-PCR reactions were performed with equal amounts of cDNA samples from all egg-laying hens using a thermal cycler (Bio-Rad, America). Table 2 showed the primers used for PCR, which were designed using Primer Premier 5 software. The specificity of each primer was checked using the NCBI blast program. The expression levels of TLRs, MDA5, and proinflammatory cytokines and chemokines were normalized to chicken β-actin and amplification efficiency were consistent with β-actin. The results of real-time PCR were quantified by the comparative threshold analysis after deductions of data from uninfected chickens.

**Table 2:** PCR primers used for mRNA expression analysis

Genes	Primer sequences	Acession no.	Location	Product size
IL-2	F: 5′-CTGTATTTCGGTAGCAATG-3′	NM_204153.1	87-247	161
	R: 5′-ACTCCTGGGTCTCAGTTG-3′			
IL-1β	F: 5′-ACCCGCTTCATCTTCTACCG-3′	NM_204524.1	663-836	174
	R: 5′-TCAGCGCCCACTTAGCTTG-3′			
IL-6	F: 5′-GGCATTCTCATTTCCTTCT-3′	NM_204628.1	853-1051	199
	R: 5′-CTGGCTGCTGGACATTTT-3′			
IFN-α	F: 5′-GGACATGGCTCCCACACTAC-3′	XM_004937097.1	2896-3044	149
	R: 5′-GGCTGCTGAGGATTTTGAAGA-3′			
IFN-β	F: 5′-CACCACCACCTTCTCCT-3′	NM_001024836.1	66-321	256
	R: 5′-TGTGCGGTCAATCCAGT-3′			
CXCLi2	F: 5′-CATCATGAAGCATTCCATCT-3′	NM_205498.1	124-223	100
	R: 5′-CTTCCAAGGGATCTTCATTT-3′			
CXCLi1	F: 5′-AACTCCGATGCCAGTG-3′	NM_205018.1	176-380	205
	R: 5′-TTGGTGTCTGCCTTGT-3′			
CCR5	F: 5′-GTGGTCAACTGCAAAAAGCA-3′	NM_001045834.1	1126-1315	190
	R: 5′-GCCCGTTCAACTGTGTCG-3′			
MDA-5	F: 5′-GGACGACCACGATCTCTGTGT-3′	NM_001193638.1	457-535	79
	R: 5′-CACCTGTCTGGTCTGCATGTTATC-3′			
TLR3	F: 5′-GCTATTGAGCAAAGTCGAGA -3′	NM_001011691.3	2491-2693	203
	R: 5′-ACAGGGGGCACTTTACTATT-3′			
TLR7	F: 5′-TCTGGACTTCTCTAACAACA-3′	NM_001011688.2	1824-2010	187
	R: 5′-AATCTCATTCTCATTCATCATCA-3′			
TLR21	F: 5′-AGTTGTGTCCTGTGCTGAGAG-3′	NM_001030558.1	1035-1164	130
	R: 5′-AGCAGGTTGTGTTCCACTGTC-3′			
β-actin	F: 5′- AGACATCAGGGTGTGATGGTTGGT-3′	NM_205518.1	183-300	118
	R: 5′-TGGTGACAATACCGTGTTCAATGG-3′			

### Detection of T lymphocytes in the magnum by immunofluorescence

The dewaxing and rehydrating process and BSA treatment was performed as described in the HN protein detection section. After removing the blocking solution, tissue sections were incubated for 1 h at 37 °C with 1 mg/ml mouse monoclonal antibody specific for chicken CD markers, FITC conjugated CD3, CY5 conjugated CD4, or RPE labeled CD8α (Southern biotech, USA) to detect CD3CD4 and CD3CD8α glycoprotein of lymphocytes. Following steps were performed as described in the HN protein detection section.

### Statistical analyses

The data are presented as the means ± SEM of three independent experiments with three replicates per experiment. Analysis of the gene expression levels was performed using one-way analysis of variance with Dunnett’s post-test in GraphPad Prism version 5.0 for Windows. *P* values less than 0.05 were considered statistically significant.
